# Effects of Sex and Obesity on *LEP* Variant and Leptin Level Associations in Intervertebral Disc Degeneration

**DOI:** 10.3390/ijms232012275

**Published:** 2022-10-14

**Authors:** Hsing-Hong Chen, Hsien-Ta Hsu, Mei-Hsiu Liao, Ming-Sheng Teng

**Affiliations:** 1 Division of Neurosurgery, Taipei Tzu Chi Hospital, Buddhist Tzu Chi Medical Foundation, New Taipei City 23142, Taiwan; 2 School of Medicine, Buddhist Tzu Chi University, Hualien 97004, Taiwan; 3 Department of Research, Taipei Tzu Chi Hospital, Buddhist Tzu Chi Medical Foundation, New Taipei City 23142, Taiwan

**Keywords:** intervertebral disc degeneration, leptin, *LEP*, single-nucleotide polymorphisms, interaction analysis

## Abstract

Intervertebral disc degeneration (IVDD), for which obesity and genetics are known risk factors, is a chronic process that alters the structure and function of the intervertebral discs (IVD). Circulating leptin is positively correlated with body weight and is often measured to elucidate the pathogenesis of IVD degeneration. In this study, we examined the associations of *LEP* single nucleotide polymorphisms (SNPs) genetic and environmental effects with IVDD. A total of 303 Taiwanese patients with IVDD (mean age, 58.6 ± 12.7 years) undergoing cervical discectomy for neck pain or lumbar discectomy for back pain were enrolled. Commercially available enzyme-linked immunosorbent assay (ELISA) kits measured the circulating plasma leptin levels. TaqMan SNP genotyping assays genotyped the *LEP* SNPs rs2167270 and rs7799039. Leptin levels were significantly increased in obese individuals (*p* < 0.001) and non-obese or obese women (*p* < 0.001). In the dominant model, recoded minor alleles of rs2167270 and rs7799039 were associated with higher leptin levels in all individuals (*p* = 0.011, *p* = 0.012). Further, the association between these *LEP* SNPs and leptin levels was significant only in obese women (*p* = 0.025 and *p* = 0.008, respectively). There was an interaction effect between sex and obesity, particularly among obese women (interaction *p* = 0.04 and 0.02, respectively). Our findings demonstrate that these SNPs have sex-specific associations with BMI in IVDD patients, and that obesity and sex, particularly among obese women, may modify the *LEP* transcription effect.

## 1. Introduction

Disc degeneration is a multifaceted chronic process that alters the structure and function of intervertebral discs (IVD) [1]. Degenerated discs occur in 40% of individuals under 30 years of age, and in more than 90% of those over 50 [2]. IVD degeneration (IVDD) may lead to disc herniation, radiculopathy, myelopathy, spinal stenosis, and/or degenerative spondylolisthesis, and can cause acute or chronic pain. IVDD manifests as different cellular and biochemical alterations, including degradation of the extracellular matrix (ECM), the buildup of cellular waste products, and an increase in the expression of pro-inflammatory cytokines [3]. Risk factors for IVDD include aging, genetics, nutrition, toxins, metabolic disorders, low-grade infections, neurogenic inflammation, autoimmune diseases, and mechanical factors [4]. Obesity is a prevalent condition in both middle- and high-income countries, and results from genetic and environmental factors. It is recognized as a systemic inflammatory state mediated by adipokines [5] and is also a known mechanical risk factor for IVDD. Furthermore, IVDD is an indicator of obesity that decreases life expectancy by ≥20% of the ideal value.

Leptin is a hormone that suppresses food intake and increases energy expenditure by binding to and activating its specific receptor in the hypothalamus [6,7]. The adipokine leptin (a 16-kDa peptide hormone) was first discovered in 1994 by Zhang et al. [8]. Leptin is released from white adipose tissue (WAT), and circulating leptin is therefore positively correlated with body fat and body mass [9,10], which increases with age and is higher in females than in males [11]. It is also commonly measured to elucidate the pathogenesis of IVDD [12,13,14,15].

Genetics is an important factor in determining the individual risk of developing disc degeneration. Traditionally, occupation, physical activity, mechanical injury, smoking, repetitive loads, gender, and vibration are dominant risk factors for accelerated degeneration [16,17,18,19,20,21]. While the influence of genetics is unclear [20], many twin studies have identified positive familial aggregation, suggesting a degree of genetic influence. In a study by Simmons et al. [22], the results showed that 44.6% of patients who underwent surgery for degenerated discs had a positive family history of degeneration, compared to 25.4% who did not. Sambrook et al. reported similar results in a study involving 172 monozygotic and 154 dizygotic twins [23]. This suggests that in addition to environmental risk factors, genetics is also an important factor in determining disc degeneration variation, which implies that disc degeneration development is possibly determined by a complex combination of factors, with gene–environment and gene–gene interactions that uniquely determine the degeneration progression in each individual [24]. In this study, we examined whether the genetic and environmental effects of *LEP* SNPs are associated with IVDD.

## 2. Results

### 2.1. Clinical and Biochemical Characteristics

Among the 303 IVDD patients recruited for analysis, the results of the association of age, BMI, smoking, and leptin levels demonstrated that lower smoking frequencies and higher leptin levels were observed in women (Table 1). Furthermore, the obesity statuses showed that leptin levels were significantly higher in obese individuals (19.91 ± 19.11) compared with non-obese individuals (*p* < 0.001); in non-obese women (10.19 ± 7.52) compared with non-obese men (*p* < 0.001); and in obese women (26.59 ± 21.54) compared with obese men (*p* < 0.001). In the baseline characteristics regarding sex and obesity of the study subjects, the frequency of smoking was significantly lower in non-obese women (Table 2).

### 2.2. Associations of LEP Polymorphisms with Respective Circulation Levels in Different Sex and Obesity Statuses

The association analyses in the additive and dominant models were adjusted for age, sex, BMI, and smoking status according to the different group selections. In the dominant model, the recoded minor alleles of rs2167270 (GA + AA) and rs7799039 (AG + GG) were associated with higher leptin levels in all individuals (12.87 ± 17.25, *p* = 0.011 and 12.28 ± 16.42, *p* = 0.012). The levels among women were 19.93 ± 21.42, *p* = 0.049 and 19.05 ± 20.48, *p* = 0.02, respectively (Table 3). We further stratified the individuals into four groups to analyze the association between *LEP* polymorphisms and leptin levels with respect to sex and obesity status. A subgroup analysis of sex according to obesity status showed that in the dominant model, the associations between *LEP* polymorphisms and leptin levels were significant only among obese women (rs2167270, 33.25 ± 27.83, *p* = 0.025; rs7799039, 33.23 ± 26.85, *p* = 0.008; Table 4). When subgroup analysis was performed on obesity according to sex, the associations between *LEP* polymorphisms and leptin levels were also only significant among obese women in the dominant model (Supplemental Table S1).

### 2.3. Interaction Analysis

**Figure 1:**
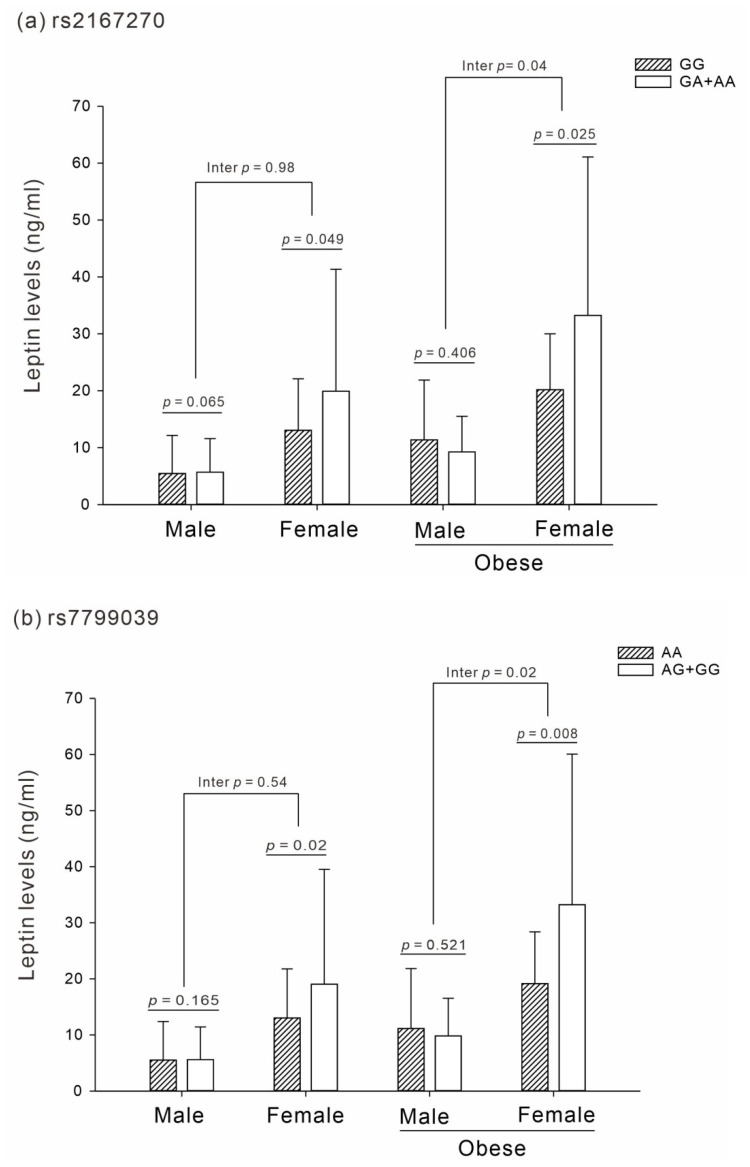
Association and interaction analysis between *LEP* SNPs and leptin levels in sex according to obesity status (*p* adjusted for age and smoking status). (**a**) Interaction effect of rs2167270 (*p* = 0.04). (**b**) Interaction effect of rs7799039 (*p* = 0.02).

The results demonstrated an interaction effect between sex and obesity within the *LEP* polymorphism and leptin level association in the dominant model of rs2167270 and rs7799039, particularly among obese women (interaction *p* = 0.04 and 0.02, respectively; Figure 1a,b). However, there was no significant interaction effect in the group of obesity according to sex (interaction *p* = 0.46 and 0.28, respectively; Supplemental Figure S1a,b).

## 3. Discussion

Our study provides evidence that leptin levels are associated with *LEP* SNPs in patients with IVDD, particularly among obese women. Leptin is an adipokine found in adipose tissue and is mostly produced in white adipose tissue, although it is also produced elsewhere such as in IVD cells [14,25,26,27,28]. Leptin has pleiotropic functions, contributes to obesity-associated chronic low-grade inflammation, and plays an important role in IVDD pathophysiology [29].

Leptin plays an important role in innate immunity, and has been shown to have direct pro-inflammatory and catabolic effects on cartilage in experimental models. It also stimulates the production of pro-inflammatory cytokines, which mediate the signaling of, inter alia, macrophages, monocytes, and dendritic cells. Several studies have demonstrated that leptin and IVDD are influenced by both sex and obesity. Krishnamoorthy et al. used a dietary mouse model to test the hypothesis that chronic consumption of diets high in advanced glycation end-products (AGEs) results in sex-specific IVD structural disruption and functional changes. They found that a high-AGE diet resulted in AGE accumulation in IVDs, as well as increased IVD compressive stiffness, decreased torque range, and increased torque failure, particularly in females [30]. Natelson et al. used in vivo diabetic and dietary mouse models to investigate whether obesity and type 2 diabetes result in spinal pathology in a sex-specific manner. They found that obesity and diabetes due to impaired leptin signaling contribute to pathological changes in the vertebrae as well as an immature IVD phenotype, particularly in females, suggesting a sex-dependent role of leptin [31]. Our previous study on IVDs from 182 patients with IVDD (mean age, 57 years) demonstrated that BMI was positively correlated with the histologic degeneration score, the plasma leptin level, and the ratio of leptin and MMP-1 immunostaining grade [32].

The etiology of IVDD as a multifactorial disease includes genetic predisposition and exposure to environmental factors, of which genetic predispositions over the past decade have been shown to be more dominant. While certain genetic factors of IVDD have been identified, most of them are unknown [33,34,35,36], and thus, the genetic mechanisms underlying IVDD remain poorly understood. Genetic modulations associated with human disc degeneration or back pain are separated by gene function, such as structural enzymes that cleave extracellular-matrix molecules and inflammatory mediators [37]. In fact, many SNPs have been reported in several studies to be associated with IVDD, such as *MMP1* and *MMP3* in the degeneration of different matrix components [38]; *COL1, 2, 9, and 11* in the degradation or loss of collagen [33,39,40,41,42]; *VDR and CLIP* in the degradation or loss of proteoglycan [43,44]; and *IL-1, IL-6 and COX2* in the increase of inflammation [45,46].

Herein, we found that two *LEP* SNPs were associated with leptin levels in patients with IVDD, which had not been reported prior to our study. However, as described above, leptin is strongly associated with IVDD because of its role in pro-inflammation [30,31,32]. Moreover, the two SNPs identified in our study had previously been extensively reported to be strongly associated with diabetes mellitus (DM) and obesity [47,48,49,50,51]. *LEP* rs7799039 promoter polymorphism is close to the specificity protein-1 (SP-1) transcription factor binding site [52]. Hoffstedt et al. reported that nuclear extracts derived from human adiposities could bind a DNA fragment spanning the 2548G/A polymorphic site [53]. Thus, it is possible that leptin rs7799039 polymorphism could affect *LEP* gene transcription and expression, thereby affecting leptin synthesis and secretion from adipose tissue [53,54]. Aly et al. conducted a study to assess the potential role of leptin and its polymorphisms as predictive markers of diabetes associated with obesity. They concluded that increased leptin levels could predict insulin resistance in obese patients. Moreover, obese subjects with the mutant genotype *LEP* gene (rs2167270) G > A showed a significantly higher susceptibility rate for DM than those with the wild type [47]. Dasgupta et al. evaluated the association between obesity and leptin gene polymorphisms and levels in a South Indian population. They found that the *LEP* SNPs rs7799039 and rs2167270 were independently and significantly associated with the risk of obesity [51]. 

DM and obesity are common risk factors for IVDD. Our findings imply that *LEP* SNPs may affect IVDD due to the mechanical stress of obesity and leptin expression induced by inflammation in adipose tissue. Interestingly, our interaction analysis found that obesity status and sex had a significant interactive effect in *LEP* SNPs related to leptin levels in patients with IVDD. We previously reported that the *LEP* SNPs rs7799039 and rs2167270 were significantly associated with leptin levels in obese women. Further univariate analysis demonstrated that both *LEP* SNPs and inflammation markers, such as CRP and E-selectin, are independently associated with leptin levels [55]. Moreover, Bains et al. conducted a case–control study in an Indian population, and reported that rs7799039 significantly increased the risk of DM in females with a BMI ≥ 23 [48]. Pawlik et al. also examined the association between leptin gene polymorphisms and the development of gestational diabetes mellitus, and found that the *LEP* rs2167270 A allele was significantly associated with GDM in women [50].

In this study, we observed a relationship between leptin variants and IVDD risk among women, which raised new questions regarding the mechanisms by which leptin and leptin gene variants might affect IVDD pathways. The mechanisms underlying sex heterogeneity observed in the aforementioned studies remain unclear. However, there are two factors that provide possible explanations. Firstly, there are varying adipokine levels between sexes, where women have higher levels of adiponectin and leptin compared to men, which thereby likely contributed to the null finding among women [56]. Secondly, the body fat distribution variations between the sexes may affect leptin levels and their effects on IVDD risk. Moreover, previous studies have suggested that sex-specific leptin levels and IVDD risk associations may be involved in the functional cross-talk between leptin and estrogen systems [31].

Several studies have described the sex-specific distribution of adipose tissue. Females have more abundant subcutaneous white adipose tissue (sWAT), whereas males have more abdominal-visceral depots [57,58]. sWAT is mainly located in the gluteal and femoral regions, and is associated with optimal metabolic health. For instance, sWAT expansion in humans is linked with improved insulin sensitivity, diminished lipolysis rate, decreased circulation of cytokines, and augmented levels of adipokines [59]. Notably, the protective role of sWAT in females seems to be age-dependent, as postmenopausal women suffer from fat redistribution where the fat depots from subcutaneous regions are transferred to the visceral regions [60]. Thus, sex hormones may play a critical role in sex-specific fat distribution and overall metabolic health. Among the sex hormones, estrogen is particularly important, as it has been shown that its decreased circulation contributes to increased adiposity, insulin resistance, low metabolic rate, and adipose tissue inflammation [61,62,63]. Moreover, the decrease in estrogen levels that comes with menopause is associated with higher risk of metabolic complications and body weight gain. Such anti-obesity effects of estrogen and its role in energy homeostasis are well established. Although Hong et al. demonstrated that male mice, compared to female mice, were more susceptible to increased body fat [62], other studies have shown that women tend to experience weight gain after menopause [64]. Estrogen has also been reported to be a key determinant of serum leptin levels and central leptin sensitivity. In diabetic Akita female mice carrying the Ins2 mutation, ERa ablation was found to exacerbate hyperphagia by further decreasing central leptin signals and downregulating POMC gene expression [65]. OVX rats displayed dramatic increases in serum leptin levels, which were associated with significant changes in body weight gain. After E2 replacement, the serum leptin levels decreased [66,67]. Both ob/ob and db/db mice treated with E2 for four weeks showed body weight loss, diminished fat mass, hypophagia, and energy expenditure. This was accompanied by elevated hypothalamic pSTAT3 levels and increased POMC immune reactivity [68]. These obesity markers were also significantly correlated with serum leptin levels, which suggests that circulating leptin could mediate the regulatory effects of estrogen signaling on adipose tissue homeostasis [69]. Similarly, estrogen has been demonstrated to have an effect on BAT thermogenesis, thermoregulation, cold adaptation, and energy expenditure [63,70,71]. Therefore, we suggest that the association among obese women of the *LEP* SNPs rs2167270 and rs7799039 with higher leptin levels in IVDD risk may be due to the post-menopausal loss of estrogen exposure and the loss of estrogen’s protective function in the downregulation of leptin expression.

There were several limitations to our study. Firstly, there was a relatively low number of genotyped subjects, and thus, replication of our results in a second cohort would support the strength of the study. Furthermore, independent association studies with larger sample sizes would not only corroborate our results, but would also allow for more definitive conclusions to be drawn. Secondly, the study sample included only Taiwanese individuals, and thus, the results cannot be generalized to other ethnicities. Given the variability of *LEP* variants and leptin levels between ethnicities, further research on different populations is required.

## 4. Materials and Methods

### 4.1. Study Population

This prospective study was approved by the local research ethics committee (IRB No. 05-XD31-061). Informed consent was obtained from all patients. During the period from March 2017 to June 2018, this study enrolled a total of 303 cervical and lumbar patients (mean age, 58.6 ± 12.7 years) undergoing cervical discectomy for neck pain with pain radiating to the upper limbs, or treated with lumbar discectomy for back pain with radicular pain to the legs. The exclusion criteria were fracture of the spine, spinal stenosis, spondylolisthesis, malignancies involving the spine, and poliomyelitis. The criteria for IVD degeneration were: (1) neck or back pain requiring visits to a physician; (2) pain problems that hampered or prevented daily activities; and (3) multiple episodes of pain. BMI ≥ 25 kg/m^2^ was classified as obese.

### 4.2. Enzyme-Linked Immunosorbent Assay

Venous blood was collected in the morning after an overnight fast. Plasma samples were obtained via centrifugation at 3000× *g* for 15 min at 4 °C. Immediately after centrifugation, plasma samples were frozen and stored at −80 °C until the time of analysis. Circulating plasma levels of leptin were measured using commercially available ELISA kits (R&D, Minneapolis, MN, USA).

### 4.3. Genomic DNA Extraction and Genotyping

Genomic DNA was extracted as reported previously [72]. Two SNPs around *LEP*s, rs7799039 (HGVS nomenclature: NC_000007.13:g.127878783A > G) and rs2167270 (HGVS nomenclature: NM_000230.2:c.-39G > A), were selected. Genotyping was performed using TaqMan SNP with genotyping assays (Thermo Fisher SCIENTIFIC, Waltham, MA USA).

### 4.4. Statistical Analysis

An independent samples *t*-test was performed for continuous variables. A Chi-square test was used to analyze categorical variables. The continuous variables, expressed as mean ± standard deviation, were tested using one-way analysis of variance (ANOVA). Tests of normality were conducted for all quantitative traits. Moreover, the leptin levels were logarithmically transformed before statistical analysis to adhere to a normality assumption. A *p* of <0.05 according to a two-sided test was considered statistically significant. Linear regression coefficients with 95% confidence intervals were calculated for leptin levels and the predicted confounders. Allelic frequencies for each SNP were estimated through gene counting, and the polymorphism distribution was tested for Hardy–Weinberg equilibrium using the Chi-square test. The stratified association analysis according to sex and obesity was performed using one-way ANOVA in additive and dominant genetic models. The statistical analysis was performed using IBM SPSS Statistics (version 22; IBM) unless otherwise specified. Furthermore, we investigated the sex- and obesity-specific effects of leptin level variants. The resultant significant polymorphisms were included in the interaction analysis using a linear regression model of SVS Win32 (version 7.3.1; Golden Helix, Bozeman, MT, USA) to determine the impact of dependent variables.

## 5. Conclusions

To our knowledge, this is the first study on the possible association between *LEP* rs7799039 and rs2167270 polymorphisms within a Taiwanese population. This study found that the frequency of IVDD is expected to be higher in the A allele of rs2167270 and the G allele of rs7799039 carriers. Our previous study disclosed that BMI is significantly associated with the HDS (histological degeneration score) [32], indicating that obesity is associated with IVDD severity. In addition, serum leptin levels in patients with IVDD are significantly related to BMI. As the number of enrolled patients with IVDD increased in the present study, the association of serum leptin levels with BMI became more significant. Based on these findings, serum leptin levels may indirectly affect IVDD, suggesting a possible influence of the *LEP* variant on IVDD. It also indicated that being overweight may have a modifying effect on SNP associations, which leads to a loss of the protective effect attributed to the alleles on the studied genes. However, based on a recent review by Curic, we cannot conclude whether detrimental and beneficial effects of the *LEP* variant exist in IVDD [73]. We suggest that detailed functional studies should be performed to investigate and understand the role of these SNPs in patients with IVDD, as well as the SNPs’ association with serum leptin levels.

## Figures and Tables

**Table 1:** Baseline characteristics of study subjects.

Variable	Total	Male	Female	*p*
Number	303	160	143	
Age	58.6 ± 12.7	57.4 ± 12.7	59.9 ± 12.7	0.091
BMI	25.4 ± 4.4	25.1 ± 4.1	25.7 ± 4.7	0.232
Smoking	25.1%	35.6%	13.3%	<0.001
Leptin (ng/mL)	10.61 ± 13.28	5.56 ± 6.36	16.27 ± 16.39	<0.001

BMI: body mass index; *p*: male vs. female (unadjusted).

**Table 2:** Baseline characteristics of study subjects in relation to sex and obesity status.

		Total		Non-Obese			Obese		
	Non-obese (212)	Obese (91)	*p*	Male (122)	Female (90)	p *	Male (38)	Female (53)	p *
Age	59.26 ± 12.3	57.03 ± 13.56	0.163	58.42 ± 12.45	60.40 ± 12.07	0.247	54.24 ± 12.94	59.04 ± 13.75	0.096
BMI	23.19 ± 2.24	30.59 ± 3.89	<0.001	23.44 ± 2.34	22.88 ± 2.07	0.071	30.57 ± 3.84	30.6 ± 3.96	
Smoking	25.5%	24.2%	0.47	36.1%	11.1%	<0.001	34.2%	24.2%	0.05
Leptin (ng/mL)	6.62 ± 6.57	19.91 ± 19.11	<0.001	3.99 ± 4.15	10.19 ± 7.52	<0.001	10.59 ± 9.14	26.59 ± 21.54	<0.001

*Note*: Data are presented as mean ± standard deviation or percentage as appropriate. Obesity was defined as BMI ≥ 25 kg/m^2.^ according to the Asian criteria (WHO Expert Consultation, 2004). *p:* adjusted for age, sex, and smoking status; p *: adjusted for age and smoking status.

**Table 3:** Association of *LEP* SNPs with leptin levels in relation to sex and obesity status.

	Total	*p*	Male	p *	Female	p *	Non-Obese	*p ^#^*	Obese	*p ^#^*
rs2167270										
GG	8.85 ± 8.69 (170)	0.837	5.46 ± 6.71 (94)	0.764	13.04 ± 9.06 (76)	0.464	5.77 ± 5.02 (119)	0.605	16.04 ± 10.99 (51)	0.638
GA	13.65 ± 18.11 (117)		6.0 ± 6.28 (56)		20.67 ± 22.21 (61)		8.08 ± 8.42 (81)		26.17 ± 26.29 (36)	
AA	7.13 ± 6.54 (16)		3.97 ± 2.29 (10)		12.39 ± 8.09 (6)		5.19 ±4.09 (12)		12.93 ± 9.61 (4)	
GG	8.85 ± 8.69 (170)	0.011	5.46 ± 6.71 (94)	0.065	13.04 ± 9.06 (76)	0.049	5.77 ± 5.02 (119)	0.073	16.04 ± 10.99 (51)	0.199
GA + AA	12.87 ± 17.25 (133)		5.7 ± 5.88 (66)		19.93 ± 21.42 (67)		7.71 ± 8.04 (93)		24.85 ± 25.37 (40)	
rs7799039										
AA	8.87 ± 8.59 (148)	0.543	5.52 ± 6.87 (82)	0.451	13.03 ± 8.75 (66)	0.279	5.82 ± 5.21 (101)	0.549	15.42 ±10.63 (47)	0.755
AG	12.89 ± 17.3 (133)		5.87 ± 6.24 (66)		19.8 ± 21.5 (67)		7.68 ± 7.98 (95)		25.91 ± 25.73 (38)	
GG	8.61 ± 8.9 (22)		4.09 ± 2.09 (12)		14.04 ± 10.94 (10)		5.4 ± 3.89 (16)		17.17 ± 12.9 (6)	
AA	8.87 ± 8.59 (148)	0.012	5.52 ± 6.87 (82)	0.165	13.03 ± 8.75 (66)	0.02	5.82 ± 5.21 (101)	0.123	15.42 ± 10.63 (47)	0.08
AG + GG	12.28 ± 16.42 (155)		5.6 ± 5.83 (78)		19.05 ± 20.48 (77)		7.35 ± 7.56 (111)		24.72 ± 24.46 (44)	

*Note:* Leptin levels, means ± *SD* (*N*); *p:* adjusted for age, sex, BMI, and smoking status; p *: adjusted for age, BMI, and smoking status; *p* *^#^:* adjusted for age, sex, and smoking status.

**Table 4:** Association between *LEP* SNPs and leptin levels in sex according to obesity status.

	Non-Obese				Obese			
	Male	*p*	Female	*p*	Male	*p*	Female	*p*
rs2167270								
GG	3.44 ± 2.74 (70)	0.956	9.11 ± 5.63 (49)	0.987	11.37 ± 10.52 (24)	0.353	20.19 ± 9.81 (27)	0.829
GA	4.99 ± 5.84 (44)		11.76 ± 9.56 (37)		9.71 ± 6.67 (12)		34.41 ± 28.61 (24)	
AA	3.32 ± 1.95 (8)		8.94 ± 4.93 (4)		6.56 ± 1.86 (2)		19.29 ± 10.57 (2)	
GG	3.44 ± 2.74 (70)	0.17	9.11 ± 5.63 (49)	0.329	11.37 ± 10.52 (24)	0.406	20.19 ± 9.81 (27)	0.025
GA + AA	4.74 ± 5.45 (52)		11.48 ± 9.21 (41)		9.26 ± 6.26 (14)		33.25 ± 27.83 (26)	
rs7799039								
AA	3.46 ± 2.83 (60)	0.993	9.28 ± 5.94 (41)	0.711	11.15 ± 10.69 (22)	0.273	19.16 ± 9.23 (25)	0.820
AG	4.69 ± 5.49 (52)		11.29 ± 9.01 (43)		10.29 ± 7.08 (14)		35.02 ± 28.31 (24)	
GG	3.59 ± 1.82 (10)		8.41 ± 4.71 (6)		6.56 ± 1.86 (2)		22.47 ± 12.8 (4)	
AA	3.46 ± 2.83 (60)	0.218	9.28 ± 5.94 (41)	0.381	11.15 ± 10.69 (22)	0.521	19.16 ± 9.23 (25)	0.008
AG + GG	4.51 ± 5.08 (62)		10.95 ± 8.62 (49)		9.82 ± 6.73 (16)		33.23 ± 26.85 (28)	

*Note*: Leptin levels, means ± *SD* (*N*); *p*: adjusted for age and smoking status.

## Data Availability

The data presented in this study are available on request from the corresponding author.

## Supplementary Materials

The following supporting information can be downloaded at: https://www.mdpi.com/article/10.3390/ijms232012275/s1.
