# Supplementary material for: Effects of Sex and Obesity on LEP Variant and Leptin Level Associations in Intervertebral Disc Degeneration
Source: Int J Mol Sci. 2022 Oct 14;23(20):12275. doi: 10.3390/ijms232012275 (PMC9603873; doi:10.3390/ijms232012275)

Supplemental Table S1. Association between *LEP* SNPs and leptin levels in sex according to obesity status

	Male				Female			
	**Non-obese**	***p***	**Obese**	***p***	**Non-obese**	***p***	**Obese**	***p***
rs2167270								
GG	3.44±2.74(70)	0.956	11.37±10.52(24)	0.353	9.11±5.63(49)	0.987	20.19±9.81(27)	0.829
GA	4.99±5.84(44)		9.71±6.67(12)		11.76±9.56(37)		34.41±28.61(24)	
AA	3.32±1.95(8)		6.56±1.86(2)		8.94±4.93(4)		19.29±10.57(2)	
GG	3.44±2.74(70)	0.17	11.37±10.52(24)	0.406	9.11±5.63(49)	0.329	20.19±9.81(27)	0.025
GA+AA	4.74±5.45(52)		9.26±6.26(14)		11.48±9.21(41)		33.25±27.83(26)	
rs7799039								
AA	3.46±2.83(60)	0.993	11.15±10.69(22)	0.273	9.28±5.94(41)	0.711	19.16±9.23(25)	0.820
AG	4.69±5.49(52)		10.29±7.08(14)		11.29±9.01(43)		35.02±28.31(24)	
GG	3.59±1.82(10)		6.56±1.86(2)		8.41±4.71(6)		22.47±12.8(4)	
AA	3.46±2.83(60)	0.218	11.15±10.69(22)	0.521	9.28±5.94(41)	0.381	19.16±9.23(25)	0.008
AG+GG	4.51±5.08(62)		9.82 ± 6.73 (16)		10.95±8.62(49)		33.23±26.85(28)	

Note: Leptin levels, Means ± *SD* (*N*); *p:* adjusted for age and smoking status.

Supplemental Figure S1. Association and interaction analysis between *LEP* SNPs and leptin levels in obesity according to sex (*P* adjusted for age and smoking status)

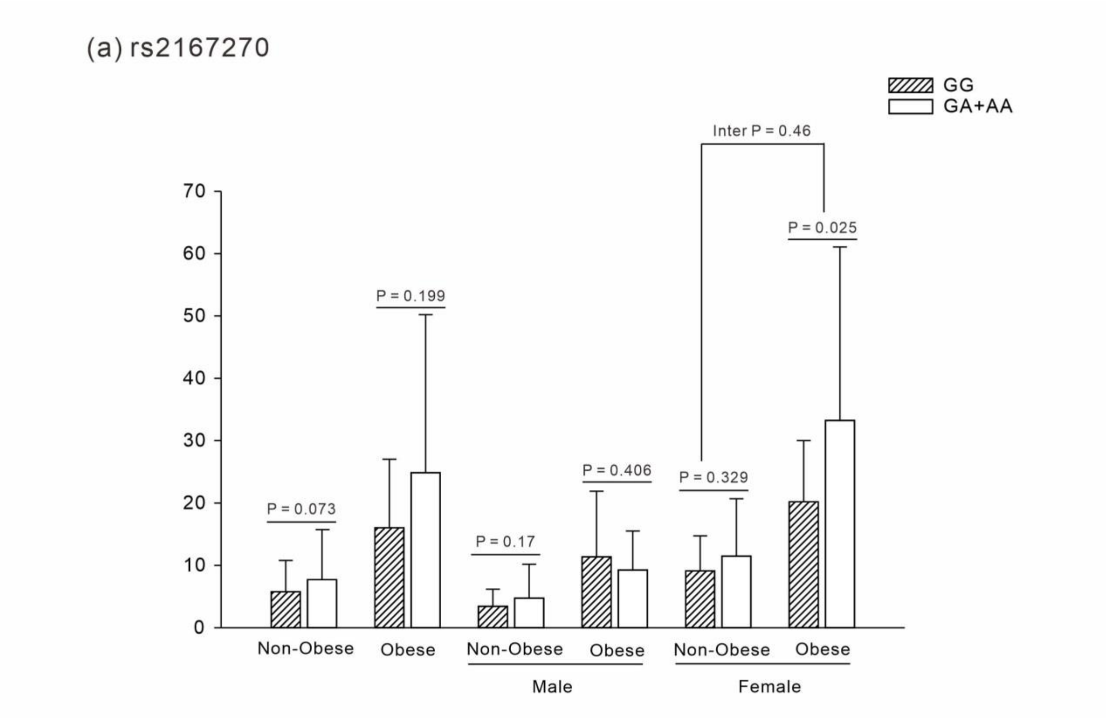

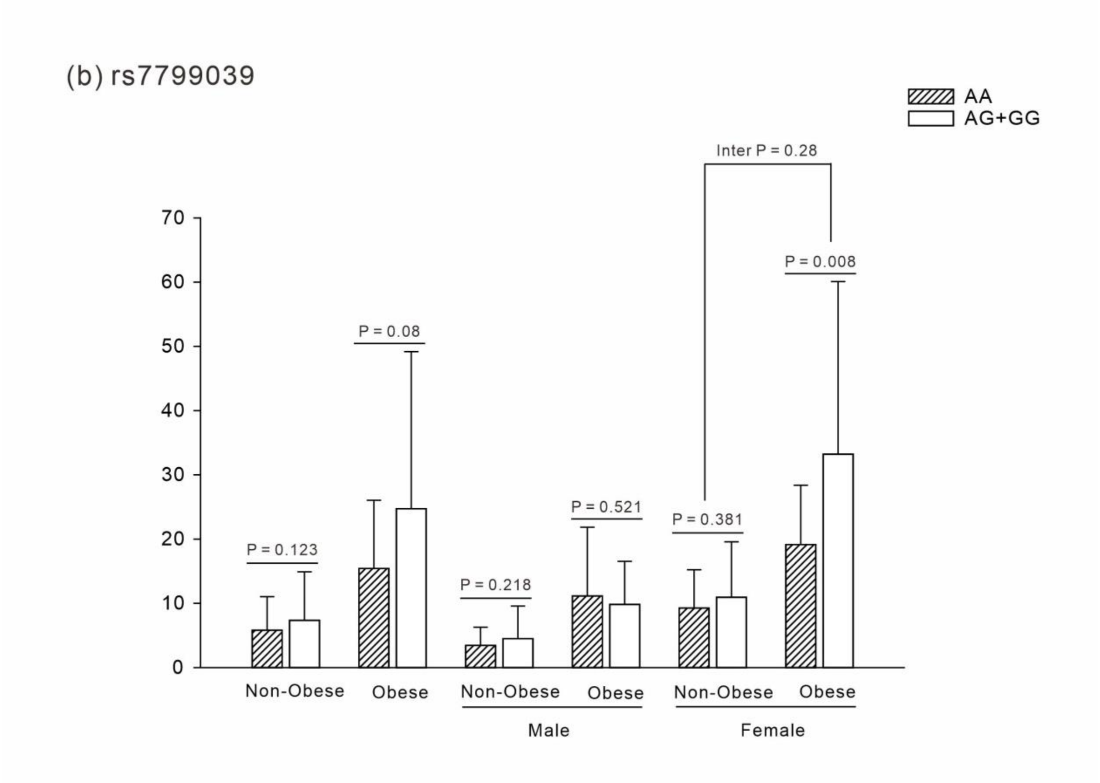

Supplement: Supplementary file 1 [file ijms-23-12275-s001.zip › ijms-1929190-supplementary.pdf]
